# An illustrated key to the genera of Thripinae (Thysanoptera, Thripidae) from Iran

**DOI:** 10.3897/zookeys.317.5447

**Published:** 2013-07-18

**Authors:** Majid Mirab-balou, Kambiz Minaei, Xue-Xin Chen

**Affiliations:** 1 Institute of Insect Sciences, Zhejiang University, 866 Yuhangtang Road, Hangzhou 310058, China; 2 Department of Plant Protection, College of Agriculture, Ilam University, Ilam, Iran; 3 Department of Plant Protection, College of Agriculture, Shiraz University, Fars, Iran

**Keywords:** Thysanoptera, Thripinae, identification, Iran

## Abstract

An illustrated key is provided for the identification of 35 genera of Thripinae (Thysanoptera: Thripidae) from Iran with comments for each genus. *Chirothrips maximi* Ananthakrishnan and *Limothrips cerealium* Haliday are recorded from Iran for the first time. A checklist is provided of Thripinae recorded from this country.

## Introduction

The family Thripidae (Thysanoptera: Terebrantia) at present comprises more than 2000 described species, which are classified into four subfamilies, Thripinae, Dendrothripinae, Sericothripinae, and Panchaetothripinae (Bhatti 1989). The present study follows the interpretation of Thripinae in this classification. Another recent classification of Terebrantia (Bhatti 2006) recognizes three superfamilies and 12 families for taxa included in the four subfamilies of Thripidae, and the taxa of the earlier subfamily Thripinae are included in three families (Chirothripidae, Projectothripidae, and Thripidae) (Bhatti 2006).

Thripinae (sensu Bhatti 1989) comprising 1600 species in 230 genera worldwide is the largest subfamily. Members of Thripinae exhibit a wide range of biologies. Many species live in flowers, on leaves, some species live in both habitats, particularly the pest species, and a few species are predators. In this subfamily, several genus-groups, which are probably monophyletic, have been recognized, including the *Anaphothrips* genus-group, the *Frankliniella* genus-group, the *Megalurothrips* genus-group, the *Scirtothrips* genus-group, the *Trichromothrips* genus-group, the *Taeniothrips* genus-group and the *Thrips* genus-group (Mound and Palmer 1981a, Mound 2002, Masumoto and Okajima 2005, 2006, 2007, Mound and Masumoto 2009).

Countries of the eastern Mediterranean comprised the most important centre for the early development of human civilization, including the development of the agricultural systems on which so much of mankind depends. In contrast, our knowledge of the natural biological systems of this area has been less actively developed. Despite excellent floristic studies, such as Flora Iranica that now provides an identification system to more than 10,000 plant species, comprehensive studies on the insect fauna of this area are sadly lacking. Iran, in particular, is a bridge between the faunas of the European and Oriental Realms, and this produces considerable difficulties in studying any single group.

Keys are available for species of some Iranian genera, such as for the genera of *Thrips* and *Frankliniella* genus-groups (Minaei et al. 2007; Mirab-balou and Chen 2011a), and the *Megalurothrips* genus-group (Mirab-balou and Chen 2011b).

Within the 35 genera of Thripinae that are now listed from Iran, many species are widely distributed and their habitats are known. The species of several genera are grass-living, including *Agalmothrips*, *Anaphothrips*, *Aptinothrips*, *Bregmatothrips*, *Chirothrips*, *Collembolothrips*, *Exothrips*, *Limothrips*, *Sitothrips*, *Sphaeropothrips*, *Stenchaetothrips*, and *Stenothrips*. On the other hand, many species especially in *Thrips* and *Frankliniella* live in various flowers, and theseinclude economic pests of agricultural crops, fruit trees, ornamental plants, greenhouses (Mirab-balou and Chen 2011a, Mirab-balou et al. 2012a). A few are predators, such as species of *Scolothrips* and *Parascolothrips*, playing an important role in checking the multiplication of tetranychid mites (Mound 2011b).

An annotated bibliography of publications on Thysanoptera of Iran was provided by Bhatti et al. (2009a). However there is not any available key to distinguish the genera. The objective of this paper is to provide an identification key to the 35 genera of Thripinae that can be recognized currently in Iran. Comments are provided for each genus. A checklist of Thripinae known from Iran are also represented here.

## Material and methods

For new records, thrips have been prepared and mounted on slides using the method of Mirab-balou and Chen (2010a) and specimens are deposited in the Institute of Insect Sciences, Zhejiang University, Hangzhou, China (**ZJUH**). All descriptions, measurements and photos were made with a Leica DM IRB microscope, a Leica MZ APO microscope with a Leica Image 1000 system. All measurements are given in micrometers (μm).

### Key to genera of Thripinae from Iran

**Table:** 

1	Pronotum without any posteroangular setae longer than discal setae (Fig. 5)	2
–	Pronotum with at least one pair of posteroangular or posteromarginal setae longer than discal setae (Figs 1–4, 6)	5
2	Antennal segments III and IV each with a simple sensorium	3
–	Antennal segments III and IV each with a forked sensorium	4
3	Antennae 9-segmented (cf. Fig. 26); apterous or macropterous; abdominal sternites without discal setae; tarsi 2-segmented; male with a transverse pore plate on abdominal sternites III–VII	*Agalmothrips*
–	Antennae 6- or 8-segmented (Fig. 29); apterous; abdominal sternites with or without discal setae; tarsi 1- or 2-segmented (tarsi 2-segmented if antennae 8-segmented, in *Aptinothrips stylifer* Trybom); male without pore plate on abdominal sternites	*Aptinothrips*
4	Median pair of setae (S1) on abdominal tergites II–VIII shorter than distance between their bases (Fig. 12); abdominal tergite VIII with comb at posterior margin	*Anaphothrips*
–	Median pair of setae (S1) on abdominal tergites II–VIII longer than distance between their bases; abdominal tergite VIII without comb at posterior margin	*Rubiothrips*
5	Abdominal tergites V–VIII each with a pair of lateral ctenidia	6
–	Abdominal tergites V–VIII without ctenidia	13
6	Abdominal tergite VIII with ctenidium situated antero-lateral to spiracle	7
–	Abdominal tergite VIII with ctenidium situated posteromesad of spiracle	9
7	Pronotum anterior margin without long setae (cf. Fig. 1); mesosternum without spinula; maxillary palp 2-segmented (cf. Fig. 20)	*Sitothrips*
–	Pronotum anterior margin with 1 or 2 pairs of setae much longer than discal setae (Fig. 6); mesosternum with spinula (cf. Fig. 15); maxillary palp 3-segmented (cf. Fig. 22)	8
8	Fore tarsus with a small tooth at apex (Fig. 23); ocellar setae pair III inserted between posterior ocelli; abdominal sternite VII of female with S1 setae inserted ahead of posterior margin (cf. Fig. 36)	*Kakothrips*
–	Fore tarsus without tooth (Fig. 21); ocellar setae pair III arising at a level ahead of posterior ocelli (Fig. 9); abdominal sternite VII of female with S1 setae arising at posterior margin (Fig. 38)	*Frankliniella*
9	Mesothoracic sternopleural sutures absent; antennal segment II without seta basad of campaniform sensillum	*Sphaeropothrips*
–	Mesothoracic sternopleural sutures present; antennal segment II with dorsal seta basad of campaniform sensillum	10
10	Prosternal basantra with several small setae; abdominal tergites at posterior margin with large triangular teeth (Fig. 45)	*Microcephalothrips*
–	Prosternal basantra without setae; posterior margin of abdominal tergites without teeth; head not distinctly smaller than pronotum	11
11	Postocular setae pair II inserted far back of the others; abdominal tergites III–V with three setae arranged straight line along the lateral margin	*Stenothrips*
–	Postocular setae pair II not displaced to the posterior although it is often slightly behind setal row; abdominal tergites III–V with three setae arranged not in straight line, median setae far from the lateral margin	12
12	Ocellar setae pair II longer than ocellar setae pair III	*Stenchaetothrips*
–	Ocellar setae pair II not longer than ocellar setae pair III (Fig. 10)	*Thrips*
13	Spinula absent on mesosternum	14
–	Spinula present on mesosternum (Fig. 15)	18
14	Abdominal tergite X of female with a pair of prominent thorn-like setae (Fig. 8); pronotum with a pair of well developed posteroangular setae. [Male apterous, with a pair of short stout setae medially on tubercles on abdominal tergite IX (Fig. 44)]	*Limothrips*
–	Abdominal tergite X of female without such thorn-like setae (Fig. 42); pronotal posteroangular setae variable	15
15	Abdominal sternite VII of female with posteromarginal setae S1 and S2 arising closer to each other than to setal pair S3	*Exothrips*
–	Abdominal sternite VII of female with posteromarginal setae arising equidistant from each other	16
16	Abdominal tergites with median pair of campaniform sensilla close to the posterior margin (Fig. 7); abdominal sternite II with three pairs of posteromarginal setae. [Male apterous, and bicolored yellow and brown (Fig. 35)]	*Bregmatothrips*
–	Abdominal tergites with median pair of campaniform sensilla arising at anterior third (cf. Figs 14, 46); abdominal sternite II with two pairs of posteromarginal setae	17
17	Pronotum broadly rectangular, with 3 pairs of posteromarginal setae; antennal segment II not prolonged laterally; fore tarsus with a curved tooth (Fig. 18); maxillary palp 2-segmented	*Collembolothrips*
–	Pronotum trapezoidal, with 5–8 pairs of posteromarginal setae (Fig. 1); antennal segment II usually projecting laterally (Fig. 30); fore tarsus without tooth (Fig. 16); maxillary palp 3-segmented	*Chirothrips*
18	Metasternum with prominent spinula	19
–	Metasternum without spinula	24
19	Antennae 6-segmented (cf. Fig. 29)	*Drepanothrips*
–	Antennae 7- or 8-segmented (Figs 27–28)	20
20	Antennae 7-segmented, VII slightly longer than VI; tarsi 1-segmented (cf. Fig. 17)	*Parascolothrips*
–	Antennae 8-segmented; tarsi 2-segmented	21
21	Pronotum with six pairs of very long setae (Fig. 2)	*Scolothrips*
–	Pronotum with no more than two pairs of elongate setae	22
22	Abdominal tergites without numerous microtrichia occupying lateral thirds, rarely with a few microtrichia near lateral margins; maxillary palp 2-segmented (cf. Fig. 20)	*Psilothrips*
–	Abdominal tergites with lateral thirds fully covered with numerous microtrichia (Fig. 37); maxillary palp 3-segmented (cf. Fig. 22)	23
23	Pronotum with two pairs of prominent posteroangular setae; antennal segment I with pair of dorsoapical setae; males of some species with antennal segment VI three times as long as segment V (Fig. 40)	*Mycterothrips*
–	Pronotum with closely spaced lines of sculpture, without long setae, or only one pair of prominent posteroangular setae (Fig. 4); antennal segment I without dorsoapical setae; antennae not sexually dimorphic	*Scirtothrips*
24	Spiracles on abdominal tergite VIII with area of specialised sculpture extending to antecostal ridge	*Chaetanaphothrips*
–	Spiracles on tergite VIII without extensive area of specialised sculpture	25
25	Head with 4 or more pairs of ocellar setae	*Florithrips*
–	Head with 2 or 3 pairs of ocellar setae	26
26	Setae on fore wing first and second veins very long, longest seta twice as long as wing width	*Euphysothrips*
–	Setae on fore wing first and second veins shorter, longest seta scarcely as long as wing width	27
27	Pronotum with four pairs of setae well-developed; antennae 9-segmented	*Ficothrips*
–	Pronotum with one or two pair of posteroangular setae well-developed, with no long anteromarginals and anteroangulars setae	28
28	Pronotum with two pairs of posteroangular setae well-developed	29
–	Pronotum with one pair of posteroangular setae	33
29	Head with only one pair of anteocellar setae (ocellar seta pair I absent) (cf. Fig. 10)	*Taeniothrips*
–	Head with two pairs of anteocellar setae (ocellar seta pair I present) (cf. Figs 9, 11)	30
30	Sensorium on antennal segment VI with elongate base (Fig. 31); fore tibia usually with 1 or 2 claw-like processes at apex; fore tarsus often with 1 or 2 small tubercles (Fig. 19)	*Odontothrips*
–	Base of sensorium on antennal segment VI not elongate; fore tibia without tubercles	31
31	Fore wing first vein with nearly complete row of setae and short interval before two distal setae (Fig. 34); clavus with 4 or 5 veinal setae	*Megalurothrips*
–	Fore wing upper vein with setal row broadly interrupted, with 1+2 distal setae (cf. Fig. 33); clavus usually with 5 veinal setae	32
32	Antennal segment I without pair of dorsoapical setae; male with a single oval or circular pore plate on each of abdominal sternites III–VII (Fig. 43)	*Tenothrips*
–	Antennal segment I with a pair of dorsal apical setae (Fig. 32); male with numerous small pore plates on abdominal sternites III–VII (Fig. 41)	*Pezothrips*
33	Antennae 9-segmented	*Eremiothrips* [in part]
–	Antennae 7- or 8-segmented	34
34	Abdominal tergites III–VI with lines of sculpture medially; sternites without discal setae	*Oxythrips*
–	Abdominal tergites without lines of sculpture medially; sternites with or without discal setae (Fig. 39)	35
35	Head longitidunaly striate behind eyes; abdominal sternite II with 0–4 discal setae	*Tamaricothrips*
–	Head weakly striate behind eyes; abdominal sternite II without discal setae	*Eremiothrips* [in part]

**Figures 1–8.**
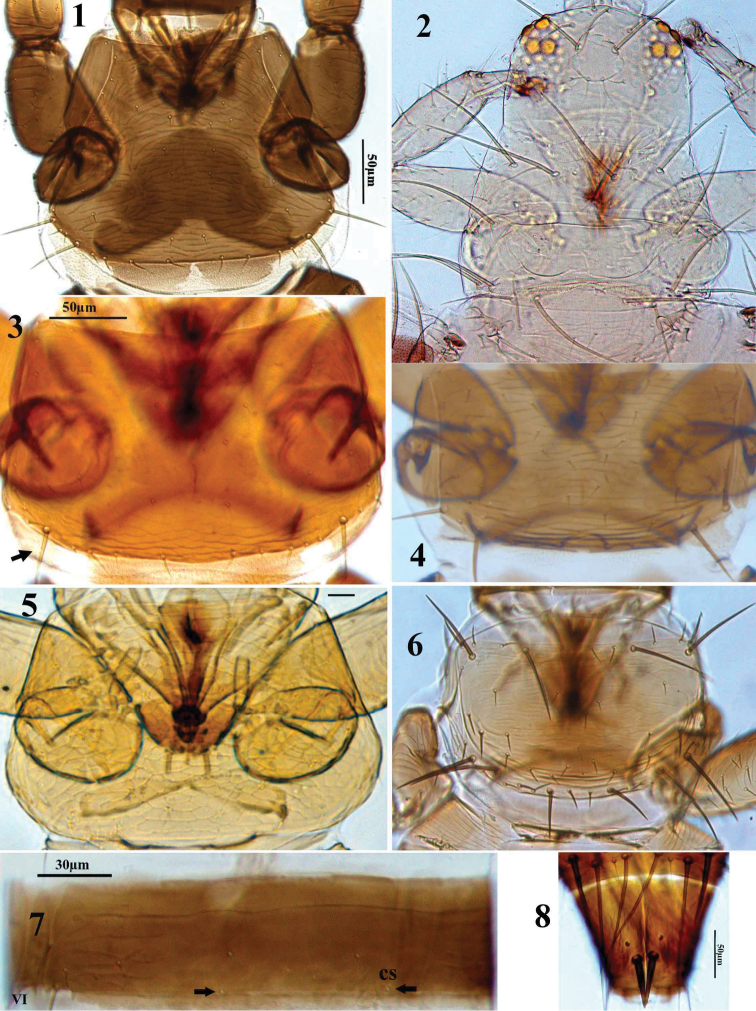
Thripinae genera. **1** *Chirothrips aculeatus*, pronotum **2** *Scolothrips longicornis* head and pronotum **3–6** pronotum **3** *Limothrips angulicornis* **4** *Tenothrips frici* **5** *Aptinothrips stylifer* **6** *Frankliniella occidentalis*, **7** *Bregmatothrips bournieri* abdominal tergite VI **8** *Limothrips angulicornis*, abdominal tergite X.

**Figures 9–15.**
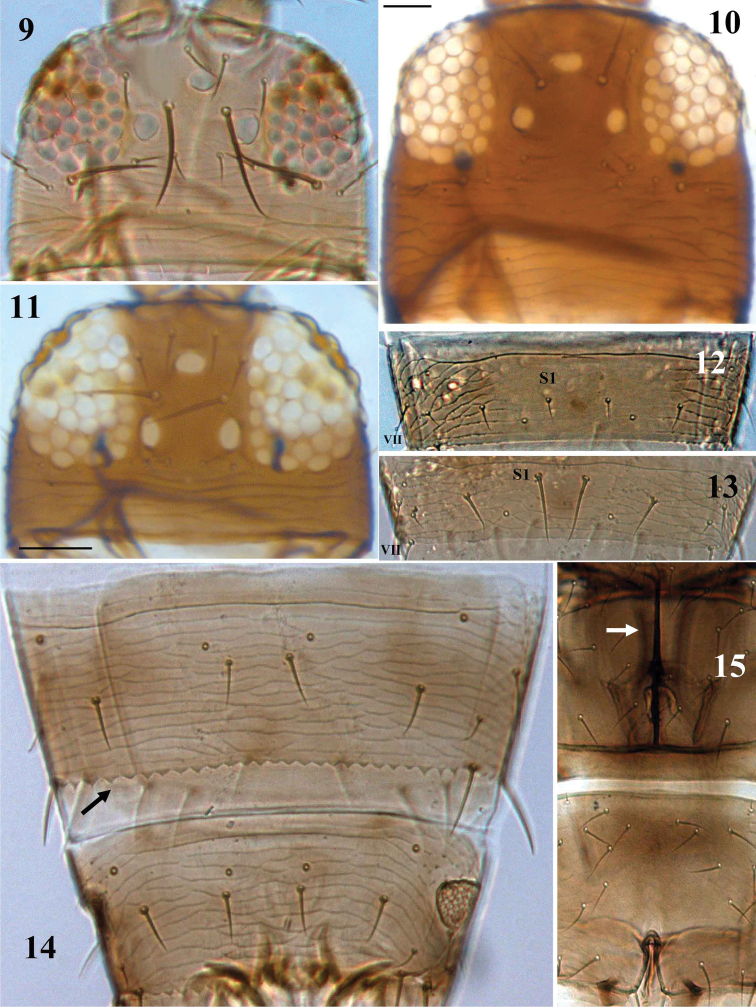
Thripinae genera. **9–11** Head, **9** *Frankliniella occidentalis* **10** *Thrips alliorum* **11** *Tenothrips frici*, **12–14** Abdominal tergite VII **12** *Anaphothrips obscurus* **13** *Rubiothrips vitis* **14** *Chirothrips aculeatus*, abdominal tergites VII–VIII, **15** *Megalurothrips distalis*, Meso- and metasterna, showing spinula.

**Figures 16–25.**
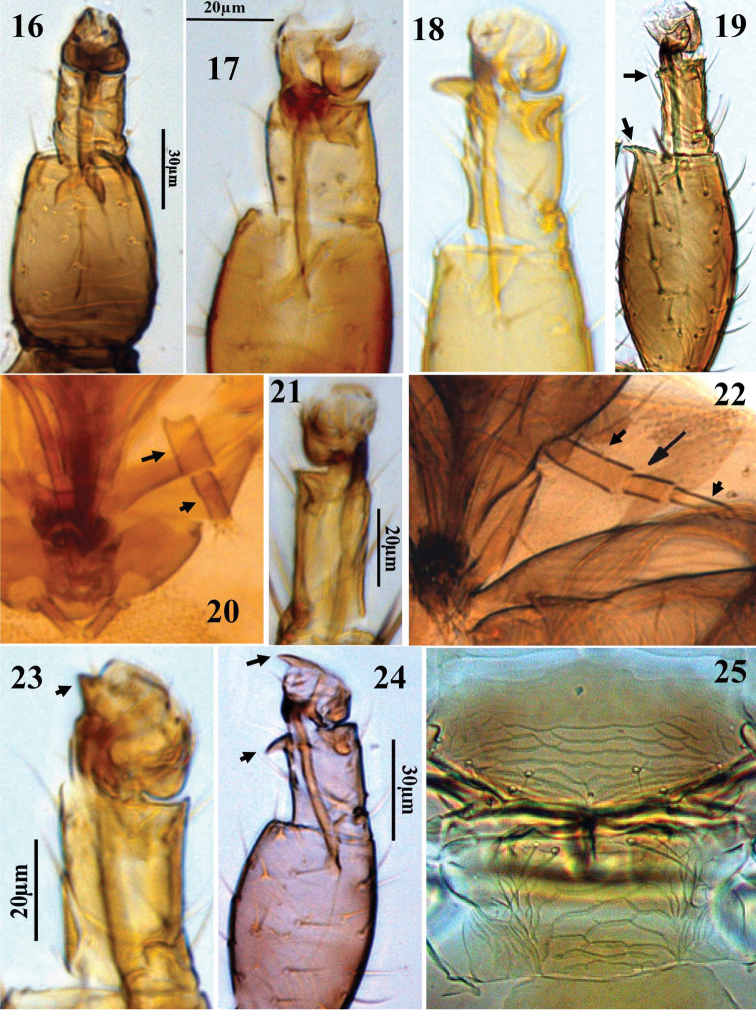
Thripinae genera. **16–19** Fore tibia and tarsus, **16** *Chirothrips aculeatus* **17** *Limothrips cerealium* **18** *Collembolothrips mediterraneus* **19** *Odontothrips loti*, **20** *Limothrips cerealium*, maxillary palps **21** *Frankliniella occidentalis*, fore tarsus **22** *Megalurothrips distalis*, maxillary palps **23–24** Fore tibia and tarsus **23** *Kakothrips pisivorus* **24** *Sitothrips arabicus* **25** *Bregmatothrips bournieri*, mesonotum and metascutum.

**Figures 26–34.**
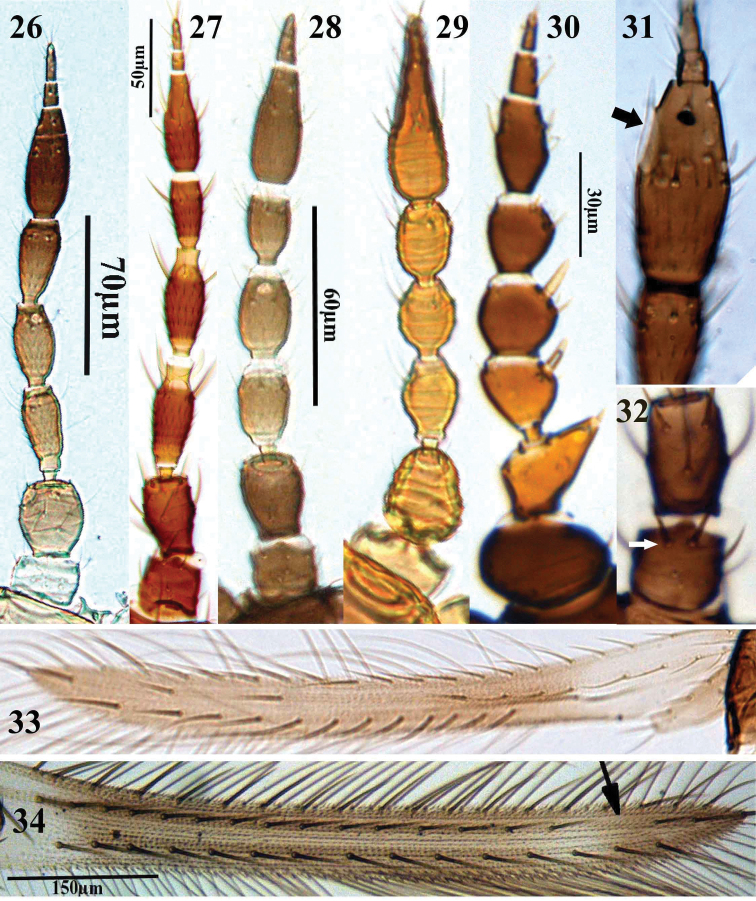
Thripinae genera. **26–32** Antennae, **26** *Anaphothrips obscurus* **27** *Pezothrips kellyanus* **28** *Microcephalothrips abdominalis* **29** *Aptinothrips elegans* **30** *Arorathrips mexicanus* **31** *Odontothrips confusus*, sensoria on segment VI **32** *Trichromothrips* sp., dorsal apical setae on segment I **33–34** Fore wing **33** *Taeniothrips inconsequens* **34** *Megalurothrips distalis*.

**Figures 35–40.**
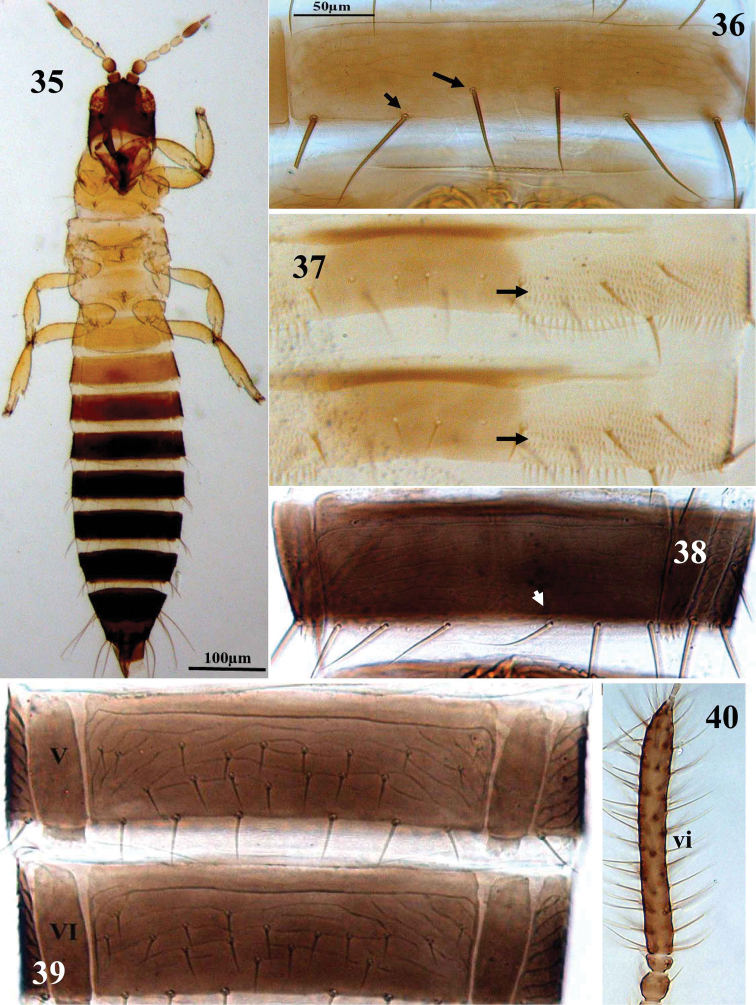
Thripinae genera. **35** *Bregmatothrips bournieri*, apterous male **36** *Pezothrips kellyanus*, abdominal sternite VII **37** *Scirtothrips dorsalis*, abdominal tergites V–VI **38** *Frankliniella tenuicornis*; abdominal sternite VII **39** *Microcephalothrips abdominalis*, abdominal sternites V–VI **40** *Mycterothrips consociatus*, antennal segment IV–VIII, male.

**Figures 41–46.**
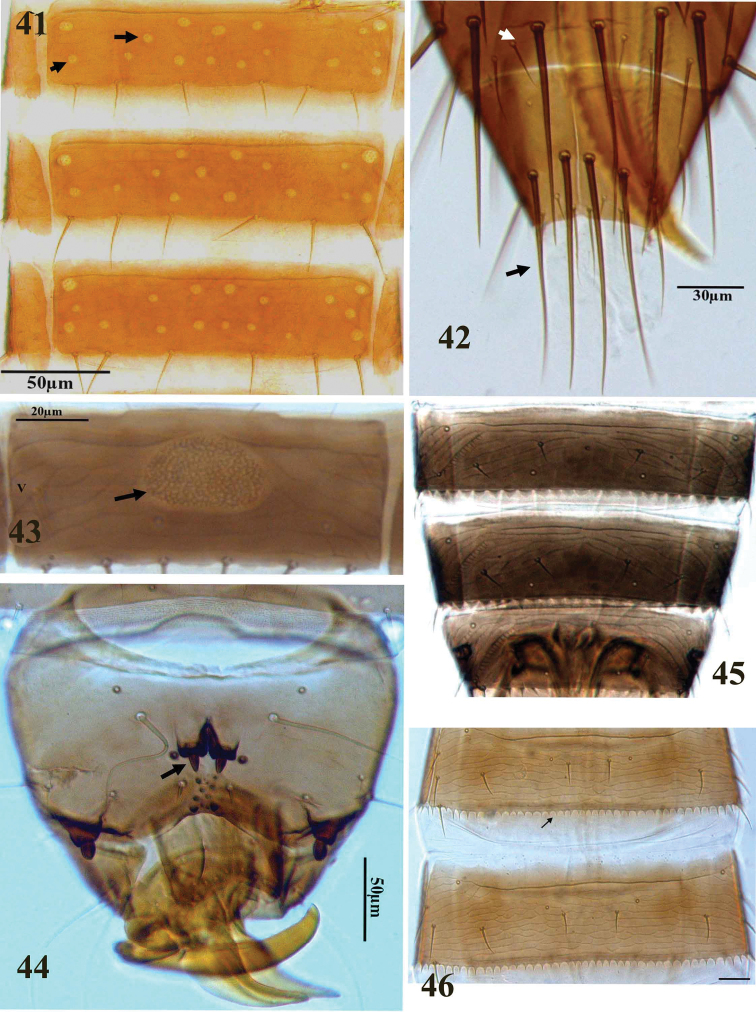
Thripinae genera. **41** *Pezothrips kellyanus*, pore plates on abdominal sternites V–VII **42** *Pezothrips kellyanus*, abdominal tergite X **43** *Tenothrips frici*, pore plate on abdominal sternite V **44** *Limothrips angulicornis*, short stout setae medially on tubercles on abdominal tergite IX **45** *Microcephalothrips abdominalis*, abdominal tergites VI–VIII **46** *Chirothrips molestus*, abdominal tergites III–IV.

#### Agalmothrips

Priesner

##### Remarks.

Included here only from descriptions, the sole species in this genus, *Agalmothrips parviceps* Priesner, was described from Sudan (Priesner 1964), subsequently reported and re-characterized from India (Bhatti 1978). It was reported from Iran by zur Strassen (2003b) based on two males and eight females collected on *Kochia* sp. (Chenopodiaceae), in Ahwaz region (Khuzestan province). *Agalmothrips* was included in the *Anaphothrips* genus-group (Mound and Masumoto 2009).

#### Anaphothrips

Uzel

##### Remarks.

This genus includes 79 species in the world (ThripsWiki 2013), and many of these are grass-living. In Iran, only *Anaphothrips obscurus* (Müller) and *Anaphothrips sudanensis* Trybom have been reported (Bhatti et al. 2009a). Recently, the male of the widely distributed species *Anaphothrips obscurus* is described only from Iran (Mirab-balou and Chen 2010b).

#### Aptinothrips

Haliday

##### Remarks.

The four species included in this genus (Mirab-balou et al. 2011a) are all apterous. Three of these are known from Iran: *Aptinothrips elegans* Priesner, *Aptinothrips rufus* (Haliday) and *Aptinothrips stylifer* Trybom (Bhatti et al. 2009a). The genus is included in the *Anaphothrips* genus-group (Mound and Masumoto 2009).

#### Bregmatothrips

Hood

##### Remarks.

This is a common genus of grass-living species in tropical and subtropical areas (Mound and Marullo 1996; Mound 2011a), with nine species worldwide (Mound 2011a, ThripsWiki 2013) of these *Bregmatothrips bournieri* Pelikán is found in Iran (Pelikán 1988; Bhatti et al. 2009a). This genus is closely related to *Sorghothrips* by having antennal segment I with paired median dorsoapical setae, and the abdominal tergites with posteromarginal craspeda and the median campaniform sensilla close to the posterior margin (Masumoto and Okajima 2006).

#### Chaetanaphothrips

Priesner

##### Remarks.

This is an Asian leaf-living genus (Pitkin 1977), and now includes 20 species (ThripsWiki 2013). Several of these are widespread around the world, and are considered pests on their host plants. *Chaetanaphothrips theiperdus* is a pest of tea in Java and Peninsular Malaysia; *Chaetanaphothrips leeuweni* (Karny), *Chaetanaphothrips orchidii* (Moulton) and *Chaetanaphothrips signipennis* (Bagnall) are pests of Bananas in some tropical countries (Kudô 1985, Mound and Marullo 1996); *Chaetanaphothrips orchidii* is a most widely distributed polyphagous species and is also recorded as a pest of some ornamental plants in greenhouses in Europe and North America (Kudô 1985, Mound and Ng 2009). Species of this genus may be distinguished from other Thripinae by the presence of modified spiracles on abdominal tergite VIII. This genus was reported from Iran by Esmaili (1983), based on an undetermined species (*Chaetanaphothrips* sp.) noted as pest of citrus fruits in Iran.

#### Chirothrips

Haliday

##### Remarks.

Species of this genus breed only in the flowers of grasses and are readily recognized from the shape of the head, pronotum, fore legs and antennae. This genus includes 53 species in the world (ThripsWiki 2013). Six species in *Chirothrips* and two species in *Agrostothrips* Hood have been reported from Iran (Bhatti et al. 2009a) but subsquentley Minaei and Mound (2010) considered *Agrostothrips* as a synonym of *Chirothrips* and five species and one species-group (*manicatus* group) have been recognized in this country. A key also to the *Chirothrips* species from Iran was given by Minaei and Mound (2010).

#### Chirothrips maximi

Ananthakrishnan

##### Remarks.

This speciesis here recorded from Iran for the first time, based on one apterous male.Hamedan province: Hamedan, Qahavand, from *Salvia nemorosa* (Lamiaceae), 20.viii.2010, coll. M. Mirab-balou (in ZJUH). The male of this speciesis easily distinguished from other species of *Chirothrips* by having small pore plates on abdominal sternite III and IV.

#### Collembolothrips

Priesner

##### Remarks.

This genus comprises two grass-living species (ThripsWiki 2013) of which *Collembolothrips mediterraneus* Priesner has been reported from Iran (Bhatti et al. 2009a). It is easily distinguished from other thripine in Iran by the absence of a spinula on both the meso- and metathoracic furca, also the lack of ocelli and wings, and the 2-segmented maxillary palps.

#### Drepanothrips

Uzel

##### Remarks.

This monobasic genus is included in *Scirtothrips* genus-group (Mound and Palmer 1981a; Masumoto and Okajima 2007). *Drepanothrips reuteri* Uzel is easily recognized from other Iranian genera by precense of the 6-segmented antennae. It is known as a pest of grapes in some areas (Mound et al. 1976; Masumoto 2010).

#### Eremiothrips

Priesner

##### Remarks.

This genus with 18 species (ThripsWiki 2013) is included in *Anaphothrips* genus-group (Mound and Masumoto 2009). Bhatti et al. (2003) published a catalogue of the *Eremiothrips* in Iran with key for separating the nine species of this genus. Recognition of species of *Eremiothrips* based on females is extremely difficult, since the general appearance and fine structural details of females are very similar in related species. However, males can be assigned readily to species because of distinctive structural features (Bhatti et al. 2003). In Iran, the genus includes 12 species (Bhatti et al. 2009b, Ramezani et al. 2009, Minaei 2012a) and a key to Iranian *Eremiothrips* based on the male sex is available (Minaei 2012a).

#### Euphysothrips

Bagnall

##### Remarks.

This genus includes two species (ThripsWiki 2013). Both are known from India but *Euphysothrips minozzii* Bagnall has been reported from Iran (Bhatti et al. 2009a). The fore wing chaetotaxy is unique, with very long setae on the veins (Mound and Ng 2009).

#### Exothrips

Priesner

##### Remarks.

This genus comprises 18 grass feeding species (ThripsWiki 2013), and is represented in Iran by *Exothrips redox* Bhatti (Bhatti et al. 2009a). Bhatti (1975) provided an identification key to 10 species from India, and indicated that these are associated with Poaceae.

#### Ficothrips

Minaei

##### Remarks.

This genus originally described from Iran with only one species, *Ficothrips moundi* (Minaei 2012b). This monobasic genus is superficially similar to *Scolothrips* or *Parascolothrips*. Morphologically, *Ficothrips* is interesting because simultaneously bears two charchter states that have been evolved rarely in Thripidae: nine antennal segments and severall long setae on the pronotum. The species was collected on fig leaves infested by *Eotetranychus hirsti* Pritchard and Baker (Acari, Tetranychidae) and the low density of species suggest that it may be a predator on that mite (Minaei 2012b).

#### Florithrips

Bhatti

##### Remarks.

This genus includes two species in the world (ThripsWiki 2013), of these *Florithrips traegardhi* was recorded from Iran on the base of a few specimens collected on corn and wheat in Khozestan Province (Ramezani et al. 2012). Leaf damage to cereal crops is recorded for this species (Mound and Kibby 1998).

#### Frankliniella

Karny

##### Remarks.

This is a large genus of about 230 species, 90% of which are from Neotropics (Mound and Marullo 1996), with only five species recorded from Iran (Mirab-balou and Chen 2011a). The genus can be recognized by having ctenidia anterolateral to each spiracle on abdominal tergite VIII, presence of five pairs of long pronotal setae, and a complete setal row on both upper and lower veins of the fore wings (Mound and Marullo 1996). A key to Iranian species is available in Mirab-balou and Chen (2011a). The species of this genus recorded from Iran are most associated with the family Rosaceae (Mirab-balou and Chen 2011a). The reports of two species from Iran have not been accepted (Bhatti and zur Strassen 2009): *Frankliniella cephalica* (D.L. Crawford) and *Frankliniella tritici* (Fitch).

#### Kakothrips

Williams

##### Remarks.

This genus includes seven species (ThripsWiki 2013), of which three are recorded from Iran (Mirab-balou and Chen 2011a). *Kakothrips* is included in the *Frankliniella* genus-group (Mound and Palmer 1981a; Mirab-balou and Chen 2011a). This genus is distinguished from *Frankliniella* by moderately developed ctenidia laterally on abdominal tergites VI–VII whereas well-developed in *Frankliniella*, and the pronotum lacks a pair of minor setae medially on the posterior margin. In addition, males of *Kakothrips* species have a pair of stout tubercles laterally on tergite VIII, whereas none of the *Frankliniella* species has such structures (Moritz et al. 2001). Zur Strassen (2003a) provided a key to seven species from Europe; and a key to the three species in Iran is available in Mirab-balou and Chen (2011a).

#### Limothrips

Haliday

##### Remarks.

This western Palaearctic genus includes eight species (ThripsWiki 2013), of which four species have been reported from Iran (Bhatti et al. 2009a). Females of this genus can be distinguished easily from other genera of Thripinae by having abdominal tergite X with short, stout, spine-like median setae (zur Strassen 2003a, Masumoto 2010).

*Limothrips cerealium* Haliday is here recorded from Iran for the first time, based on one apterous female: Azarbaijan-e-Sharghi province: Tabriz, Miyaneh, from harvested wheat, *Triticum aestivum* L. (Poaceae), 26.vi.2009, M. Mirab-balou, (in ZJUH).

This species is distinguished from other species by tarsi 1-segmented, absence of ocelli, and antennal segments III and IV with simple sensoria. This European pest of cereal crops is now widespread throughout the temperate and subtropical areas of the world. Adults are usuallymacropterous, but apterae have been recorded from Sardinia (Karny 1914) and Corsica (Mound and Palmer 1973), and now from Iran.

#### Megalurothrips

Bagnall

##### Remarks.

Thirteen species are included in this genus (ThripsWiki 2013), all breeding in the flowers of Fabaceae some as pests of cultivated legumes (Masumoto 2010). Recently, *Megalurothrips distalis* (Karny) was recorded from Iran (Mirab-balou and Chen 2011b). The members of this genus all have a pair of dorso-apical setae on the first antennal segment, and abdominal tergite VIII with many scattered microtrichia anterior to the spiracles (Mound and Ng 2009).

#### Microcephalothrips

Bagnall

##### Remarks.

This monobasic genus is included in *Thrips* genus-group (Mound and Palmer 1981a; Mirab-balou and Chen 2011a), from which it can be distinguished by the key above. *Microcephalothrips abdominalis* lives in the flowers of various Asteraceae, particularly sunflower, *Helianthus annuus* (Palmer 1992; Mound and Marullo 1996). The macropterous morph is common in Iran. Recently one micropterous male was collected on grasses from Ilam Province, and is firstly recorded of micropterous morph for Iran.

#### Mycterothrips

Trybom

##### Remarks.

The 27 known species of *Mycterothrips* (ThripsWiki 2013) are leaf-feeding thrips, and some of them are associated with agricultural crops (Masumoto and Okajima 2006). An identification key to five species from Taiwan was provided by Wang (1999), a key to seven species has been provided by zur Strassen (2003a), and a key to 27 species by Masumoto and Okajima (2006). Up to now, four species have been recorded from Iran, with two new species described recently (Mirab-balou et al. 2011b).

#### Odontothrips

Amyot & Serville

##### Remarks.

The 31 species of this genus (ThripsWiki 2013) are typical flower-living thrips, and most are associated with plants of the family Fabaceae. They can cause slight damage to the flowers, but only *Odontothrips confusus* is an important pest (Pitkin 1972). *Megalurothrips peculiaris* which sensorium base is similar to *Odontothrips*, and recorded from India and Bangladesh. This genus is included in the *Megalurothrips* genus-group (Mound and Palmer 1981a) and four species have been recorded from Iran (Mirab-balou and Chen 2011b). *Odontothrips confusus* Priesner is widely distributed in Iran, and populations are sometimes high on alfalfa, *Medicago sativa*.

#### Oxythrips

Uzel

##### Remarks.

This genus includes 50 species (ThripsWiki 2013), of which five species are known from Iran (Bhatti et al. 2009a). *Oxythrips* is included in *Anaphothrips* genus-group (Mound and Masumoto 2009).

#### Parascolothrips

Mound

##### Remarks.

This monobasic genus with one predatory species, *Parascolothrips priesneri* Mound, has been reported from Iraq (Mound 1967, ThripsWiki 2013). Mound (1967) described the species from Iraq with three pairs of posteromarginal setae on sternites II–VI, but in Iranian specimens, there are three pairs of setae on sternite II and four pairs on sternites III–VI.

#### Pezothrips

Karny

##### Remarks.

This genus is placed in the *Megalurothrips* genus-group (Mound and Palmer 1981a; Mirab-balou and Chen 2011b) and currently includes 10 species (Mirab-balou and Tong 2012). Only one species, *Pezothrips bactrianus* (Pelikan), has been reported from Iran (Mirab-balou and Chen 2011b).

#### Psilothrips

Hood

##### Remarks.

Included here only from descriptions, this genus includes five species in the world (ThripsWiki 2013) of which *Psilothrips bimaculatus* (Priesner) has been reported from Iran (Bhatti et al. 2009a).

#### Rubiothrips

Schliephake

##### Remarks.

This genus includes seven species (ThripsWiki 2013), and one of these has been recorded from Iran (Bhatti et al. 2009a). The genus is included in the *Anaphothrips* genus-group (Mound and Masumoto 2009), and is distinguished from *Anaphothrips* by having abdominal tergites with median pair of setae (S1) longer than inter-distance (zur Strassen 2003a).

#### Scirtothrips

Shull

##### Remarks.

The genus *Scirtothrips* currently includes 103 species (ThripsWiki 2013), and two of these have been recorded from Iran (Bhatti et al. 2009a). It includes several important pest species (Mound and Palmer 1981b, Mirab-balou et al. 2012b). The report of *Scirtothrips citri* (Moulton) from Iran has not been accepted (Bhatti and zur Strassen 2009).

#### Scolothrips

Hinds

##### Remarks.

Species of the genus *Scolothrips* are well known as predators of mites on the leaves of plants. The genus is easy to recognize by the presence of six pairs of very long setae on the pronotum, and the fore wings with dark bands, although recognition of species within the genus has remained difficult (Mound 2011b). Currently 16 species are recognized (ThripsWiki 2013), of which three are reported from Iran (Bhatti et al. 2009a); but the report of *Scolothrips sexmaculatus* (Pergande) from Iran has not been accepted (Bhatti and zur Strassen 2009).

#### Sitothrips

Priesner

##### Remarks.

This genus includes four species (ThripsWiki 2013) of which *Sitothrips arabicus* Priesner has been recorded from Iran (Bhatti et al. 2009a, Mirab-balou and Chen 2011a). This genus is included in the *Frankliniella* genus-group (Mound and Palmer 1981a). It is distinguished from other members of this group by lack of long setae on the anterior margin of the pronotum, the meso- and metathoracic furcae both without a spinula, the maxillary palps 2-segmented, and the fore tarsi with two small teeth (one at the apex of the tarsus, the other at the end of the first segment) (Mirab-balou and Chen 2011a). A key to three species of *Sitothrips* is available in zur Strassen (2003a). In some parts of Iran, like as Golestan province, *Sitothrips arabicus* is largely present on wheat and barley (Alavi et al. 2007)

#### Sphaeropothrips

Priesner

##### Remarks.

This monotypic genus includes a single grass-living species, *Sphaeropothrips vittipennis* (Bagnall) that was recorded from Iran by Minaei et al. (2007). It is included in *Thrips* genus-group (Mound and Palmer 1981a).

#### Stenchaetothrips

Bagnall

##### Remarks.

This genus includes 35 species (ThripsWiki 2013) and it is associated with the plant family Poaceae. *Stenchaetothrips biformis* is widely distributed, and was recorded from Iran by Mirab-balou and Chen (2011a). The genus is included in *Thrips* genus-group (Mound and Palmer 1981a; Mirab-balou and Chen 2011a) and is closely related to *Thrips*, but it can be distinguished by the length of ocellar setae II as in the above key, and the metanotum is generally longitudinally striate (Mound and Ng 2009).

#### Stenothrips

Uzel

##### Remarks.

The only species in this genus, *Stenothrips graminum* Uzel, was recorded from Iran by Minaei et al. (2007). It is a member of *Thrips* genus-group (Mound and Palmer 1981a).

#### Taeniothrips

Amyot & Serville

##### Remarks.

This genus includes 25 species in the world (Mound et al. 2012; ThripsWiki 2013). *Taeniothrips inconsequens* (Uzel) is the only species recorded from Iran (Bhatti et al. 2009a).

#### Tamaricothrips

Priesner

##### Remarks.

Only one species is placed in this genus, is also recorded from Iran (Bhatti et al. 2009a). This species is possibly more widespread in association with *Tamarix* species (zur Strassen 2003a). The genus is included in *Anaphothrips* genus-group (Mound and Masumoto 2009).

#### Tenothrips

Bhatti

##### Remarks.

This genus includes 19 flower-living species in the world (ThripsWiki 2013), of which *Tenothrips frici* (Uzel), is widespread around the world in warm temperate areas (Mound and Marullo 1996; Mirab-balou and Tong 2013). Bhatti (2003) reviewed this genus, listing 19 species. Four species are reported from Iran (Bhatti et al. 2009a), especially on the plant family Asteraceae, and *Tenothrips frici* is widely distributed in this country.

#### Thrips

Linnaeus

##### Remarks.

This genus includes about 280 species in the world and is the largest genus in the Thripinae (ThripsWiki 2013), and includes 29 species in Iran (Mirab-balou et al. 2012a; Minaei 2012c). The genus shows extensive diversity in most parts of the world except the Neotropical region, and includes many species of economic importance (Bhatti 1980). Several species of *Thrips* are considered crop pests in various parts of the world, such as *Thrips angusticeps* Uzel, *Thrips flavus* Schrank, *Thrips hawaiiensis* (Morgan), *Thrips meridionalis* Priesner, and *Thrips tabaci* Lindeman (Moritz et al. 2001). The latter species is well known as the most important pest of onion crops, greenhouses and ornamental plants in Iran (Mirab-balou and Chen 2011a, Mirab-balou et al. 2012b), and is a vector of some Tospovirus diseases on plants.

## Supplementary Material

XML Treatment for Agalmothrips

XML Treatment for Anaphothrips

XML Treatment for Aptinothrips

XML Treatment for Bregmatothrips

XML Treatment for Chaetanaphothrips

XML Treatment for Chirothrips

XML Treatment for Chirothrips maximi

XML Treatment for Collembolothrips

XML Treatment for Drepanothrips

XML Treatment for Eremiothrips

XML Treatment for Euphysothrips

XML Treatment for Exothrips

XML Treatment for Ficothrips

XML Treatment for Florithrips

XML Treatment for Frankliniella

XML Treatment for Kakothrips

XML Treatment for Limothrips

XML Treatment for Megalurothrips

XML Treatment for Microcephalothrips

XML Treatment for Mycterothrips

XML Treatment for Odontothrips

XML Treatment for Oxythrips

XML Treatment for Parascolothrips

XML Treatment for Pezothrips

XML Treatment for Psilothrips

XML Treatment for Rubiothrips

XML Treatment for Scirtothrips

XML Treatment for Scolothrips

XML Treatment for Sitothrips

XML Treatment for Sphaeropothrips

XML Treatment for Stenchaetothrips

XML Treatment for Stenothrips

XML Treatment for Taeniothrips

XML Treatment for Tamaricothrips

XML Treatment for Tenothrips

XML Treatment for Thrips

## Appendix

### Checklist of Thripinae known from Iran

*Agalmothrips parviceps* Priesner

*Anaphothrips obscurus* (Müller)

*Anaphothrips sudanensis* Trybom

*Aptinothrips elegans* Priesner

*Aptinothrips rufus* (Haliday)

*Aptinothrips stylifer* Trybom

*Bregmatothrips bournieri* Pelikan

*Chaetanaphothrips* sp.

*Chirothrips aculeatus* Bagnall

*Chirothrips africanus* Priesner

*Chirothrips atricorpus* Girault

*Chirothrips kurdistanus* zur Strassen

*Chirothrips manicatus* (Haliday)

*Chirothrips maximi* Ananthakrishnan

*Chirothrips meridionalis* Bagnall

*Chirothrips molestus* Priesner

*Chirothrips pallidicornis* Priesner

*Collembolothrips mediterraneus* Priesner

*Drepanothrips reuteri* Uzel

*Eremiothrips antilope* (Priesner)

*Eremiothrips arya* (zur Strassen)

*Eremiothrips bhattii* Minaei

*Eremiothrips dubius* (Priesner)

*Eremiothrips efflatouni* (Priesner)

*Eremiothrips farsi* Bhatti and Telmadarraiy

*Eremiothrips shirabudinensis* (Jaknontov)

*Eremiothrips similis* Bhatti

*Eremiothrips taghizadehi* (zur Strassen)

*Eremiothrips tamaricis* (zur Strassen)

*Eremiothrips varius* (Bhatti)

*Eremiothrips zurstrasseni* Bhatti, Bagheri, and Ramezani

*Euphysothrips minozzii* Bagnall

*Exothrips redox* Bhatti

*Ficothrips moundi* Minaei

*Florithrips traegardhi* (Trybom)

*Frankliniella intonsa* (Trybom)

*Frankliniella occidentalis* (Pergande)

*Frankliniella pallida* (Uzel)

*Frankliniella schultzei* (Trybom)

*Frankliniella tenuicornis* (Uzel)

*Kakothrips dentatus* Knechtel

*Kakothrips pisivorus* (Westwood)

*Kakothrips priesneri* Pelikan

*Limothrips angulicornis* Jablonowski

*Limothrips cerealium* Haliday

*Limothrips denticornis* (Haliday)

*Limothrips schmutzi* Priesner

*Limothrips transcaucasicus* Savenko

*Megalurothrips distalis* (Karny)

*Microcephalothrips abdominalis* (Crawford)

*Mycterothrips consociatus* (Targioni-Tozzetti)

*Mycterothrips hamedaniensis* Mirab-balou, Shi and Chen

*Mycterothrips latus* (Bagnall)

*Mycterothrips salicis* (Reuter)

*Mycterothrips tschirkunae* (Yakhontov)

*Mycterothrips weii* Mirab-balou, Shi and Chen

*Odontothrips confusus* Priesner

*Odontothrips meliloti* Priesner

*Odontothrips loti* (Haliday)

*Odontothrips phlomidinus* Priesner

*Oxythrips claripennis* Priesner

*Oxythrips halidayi* Bagnall

*Oxythrips retamae* (Priesner)

*Oxythrips ulmifoliorum* (Haliday)

*Oxythrips wiltshirei* Priesner

*Parascolothrips priesneri* Mound

*Pezothrips bactrianus* (Pelikan)

*Psilothrips bimaculatus* (Priesner)

*Rubiothrips vitis* (Priesner)

*Scirtothrips citri* (Moulton)

*Scirtothrips mangiferae* Priesner

*Scolothrips latipennis* Priesner

*Scolothrips longicornis* Priesner

*Scolothrips rhagebianus* Priesner

*Sitothrips arabicus* Priesner

*Sphaeropothrips vittipennis* (Bagnall)

*Stenchaetothrips biformis* (Bagnall)

*Stenothrips graminum* Uzel

*Taeniothrips inconsequens* (Uzel)

*Tamaricothrips tamaricis* (Bagnall)

*Tenothrips discolor* (Karny)

*Tenothrips frici* (Uzel)

*Tenothrips latoides* (Pelikán)

*Tenothrips reichardti* (Priesner)

*Thrips alavii* Mirab-balou, Tong and Chen

*Thrips alliorum* (Priesner)

*Thrips angusticeps* Uzel

*Thrips atratus* Haliday

*Thrips australis* (Bagnall)

*Thrips coloratus* Schmutz

*Thrips dubius* Priesner

*Thrips euphorbiae* Knechtel

*Thrips flavus* Schrank

*Thrips fraudulentus* (Priesner)

*Thrips fuscipennis* Haliday

*Thrips hawaiiensis* (Morgan)

*Thrips iranicus* Yakhontov

*Thrips major* Uzel

*Thrips mareoticus* (Priesner)

*Thrips meridionalis* (Priesner)

*Thrips minutissimus* Linnaeus

*Thrips nigropilosus* Uzel

*Thrips pelikani* Schliephake

*Thrips physapus* Linnaeus

*Thrips pillichi* Priesner

*Thrips pistaciae* Yakhontov

*Thrips simplex* (Morison)

*Thrips tabaci* Lindeman

*Thrips trehernei* Priesner

*Thrips trybomi* (Karny)

*Thrips verbasci* (Priesner)

*Thrips vuilleti* (Bagnall)

*Thrips vulgatissimus* Haliday
